# Estimating scrub typhus and murine typhus incidence among adolescents and adults in Yangon, Myanmar

**DOI:** 10.1111/tmi.70010

**Published:** 2025-07-22

**Authors:** Win Thandar Oo, Thomas R. Bowhay, Tin Ohn Myat, Wah Win Htike, Kay Thi Lwin, Stuart D. Blacksell, Ampai Tanganuchitcharnchai, Mayfong Mayxay, Paul N. Newton, Matthew T. Robinson, James E. Ussher, David R. Murdoch, Hla Hla Win, John A. Crump

**Affiliations:** ^1^ Department of Microbiology University of Medicine 1 Yangon Myanmar; ^2^ Centre for International Health University of Otago Dunedin New Zealand; ^3^ Department of Preventive and Social Medicine University of Medicine 1 Yangon Myanmar; ^4^ Mahidol‐Oxford Tropical Research Medicine Unit, Faculty of Tropical Medicine, Mahidol University Bangkok Thailand; ^5^ Centre for Tropical Medicine and Global Health, Nuffield Department of Medicine University of Oxford Oxford UK; ^6^ Institute of Research and Education Development (IRED), University of Health Sciences, Ministry of Health Vientiane Lao PDR; ^7^ Lao‐Oxford‐Mahosot Hospital‐Wellcome Trust Research Unit (LOMWRU), Microbiology Laboratory, Mahosot Hospital Vientiane Lao PDR; ^8^ Department of Microbiology and Immunology University of Otago Dunedin New Zealand; ^9^ Department of Pathology and Biomedical Science University of Otago Christchurch New Zealand

**Keywords:** incidence studies, murine typhus, Myanmar, scrub typhus, spotted fever group rickettsiosis

## Abstract

**Objectives:**

Rickettsioses are frequent causes of treatable febrile illness in Southeast Asia, including Myanmar. Accurate estimates of the incidence of rickettsioses are needed to inform investments in disease prevention and control. We sought to estimate the incidence of rickettsioses among adults and adolescents by combining sentinel hospital surveillance with a healthcare utilisation survey in Yangon, Myanmar.

**Methods:**

We conducted a household‐based healthcare utilisation survey in the Yangon Region from 12 March through 5 April 2018. Multipliers derived from this survey were then applied to scrub typhus, murine typhus, and spotted fever group rickettsioses infections identified from a study of adolescent and adult community‐onset febrile illness done from 5 October 2015 through 4 October 2016 at Yangon General Hospital to estimate disease incidence. Acute serum was collected at enrolment and convalescent serum 14–30 days after enrolment. Confirmed acute scrub typhus, murine typhus, and spotted fever group infections were diagnosed by a ≥ 4‐fold rise between acute and convalescent immunofluorescent antibody test titre to *Orientia tsutsugamushi* pooled Karp, Kato, and Gilliam antigens; *Rickettsia typhi* Wilmington strain; and *Rickettsia honei* and *Rickettsia conorii* antigens, respectively.

**Results:**

After applying multipliers, we estimated the overall annual incidence of acute scrub typhus among adolescents and adults in the Yangon Region at 211 cases per 100,000 persons, and the overall estimate of acute murine typhus among adults and adolescents was 44 cases per 100,000 persons per year for 2015–2016. There were no confirmed spotted fever group infections.

**Conclusions:**

We provide the first estimates of scrub typhus and murine typhus community incidence in Myanmar. Similar research in children and from other parts of Myanmar, as well as studies of illness duration, complications, and deaths, is needed to estimate the disease burden.

## INTRODUCTION

Rickettsioses are diverse vector‐borne bacterial diseases caused by organisms in the family Rickettsiaceae, including the genera *Rickettsia* and *Orientia* [1]. The *Rickettsia* genus is divided into two major antigenic groups: the typhus group (TG) including *Rickettsia typhi* causing murine typhus, and the spotted fever group (SFG) *Rickettsia* associated with spotted fever rickettsioses such as *Rickettsia conorii* and *Rickettsia honei*. The genus *Orientia* includes scrub typhus caused by *Orientia tsutsugamushi* as well as recently described *Candidatus* Orientia chuto and *Candidatus* Orientia chiloensis [2, 3, 4, 5, 6]. *O. tsutsugamushi* comprises a vast diversity of strains with considerable antigenic variability; strains Karp, Kato, and Gilliam are commonly found in Asia [7]. Rickettsial infections are frequent causes of treatable febrile illness in Southeast Asia. After excluding dengue and malaria, rickettsial infections were responsible for 24.0% of all infections in febrile hospitalised patients in Thailand [8]. Illness severity and risk for death vary, with systematic reviews showing an untreated case fatality ratio for murine typhus of approximately 0.4% and for scrub typhus of approximately 6.0% [9, 10].

Scrub typhus was documented in Myanmar during the 1940s [11], and serological evidence of scrub typhus, murine typhus, and SFG rickettsioses have been reported in central Myanmar more recently [12]. To our knowledge, there are no published estimates of the incidence of these diseases in Myanmar. Incidence estimates contribute to the burden of disease calculations that, in turn, inform decisions on prioritising investments in disease management, prevention, and control. We combined the results of the sentinel surveillance study of causes of severe febrile illness presenting to Yangon General Hospital (YGH) with those of a health care utilisation survey (HCUS) to estimate the incidence of scrub typhus, murine typhus, and spotted fever group rickettsioses to contribute to an understanding of the burden of these diseases in Yangon, Myanmar.

**FIGURE 1:**
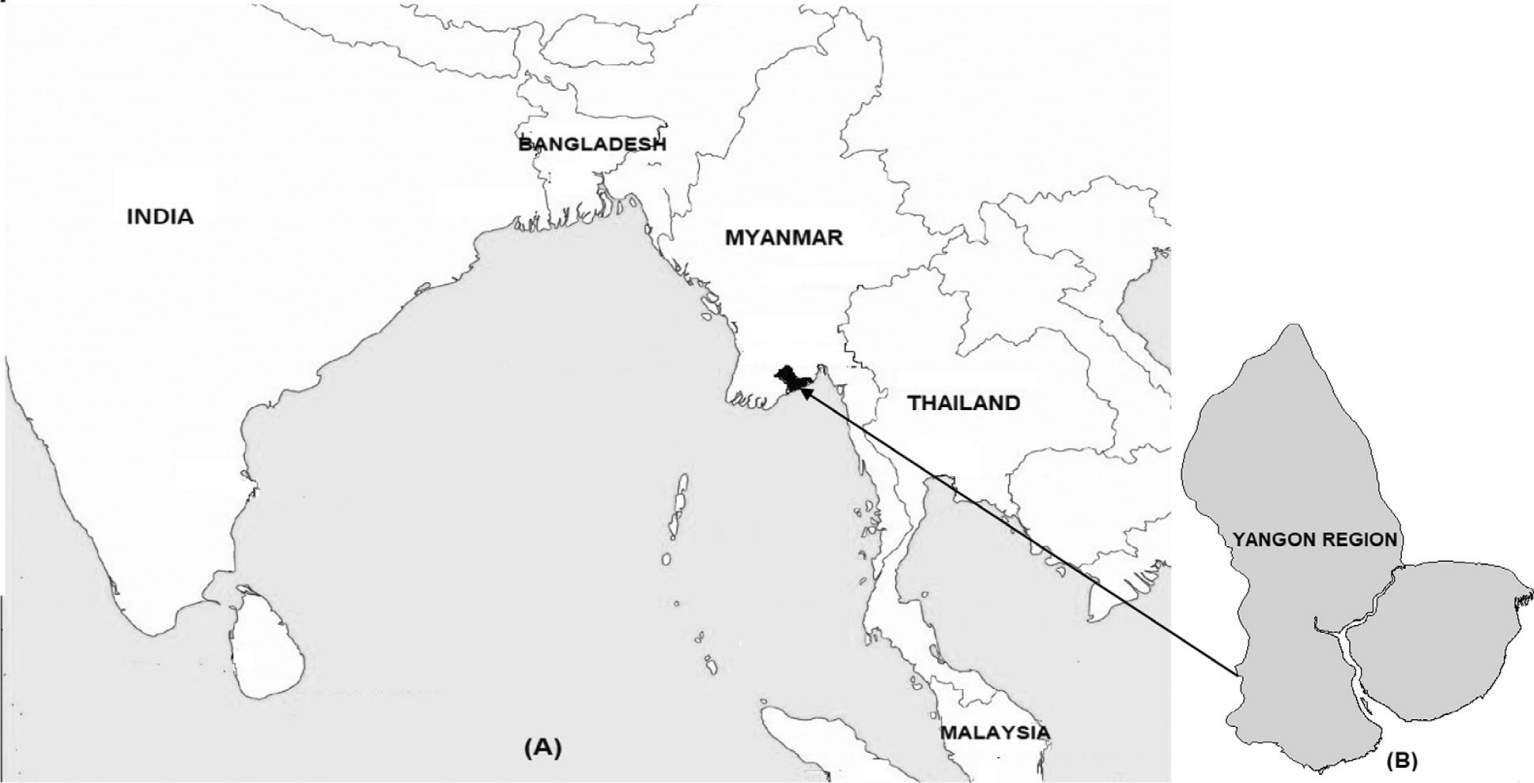
Map of South and South‐East Asia showing Myanmar (a) and Yangon Region (b).

## METHODS

### Study location

The study was performed in Yangon, Myanmar (Figure 1). The Yangon Region had a population of 7,360,703 in 2014 [13]. The Region is served by YGH, a 2000‐bed public civilian tertiary referral hospital for adolescents and adults, as well as by hospitals in each district of the Yangon Region.

### Healthcare utilisation survey

#### Household selection

We did a healthcare utilisation survey based on the World Health Organization's (WHO) two‐stage cluster survey methodology [14]. The Yangon Region included four districts encompassing 46 townships. The townships were further subdivided into 689 wards. According to sample size calculation derived from the WHO methodology, 48 of the 689 wards of the Yangon Region were randomly selected proportional to population size according to the 2014 Myanmar population and housing census in the first step [13]. In the second stage, 7 households were selected for each of the 48 wards by simple random sampling from ward household lists provided by Township Health Departments. If a household declined to participate, it was replaced by another randomly selected household.

#### Study design administration of the survey

The healthcare utilisation survey was conducted in the Yangon Region from 12 March through 5 April 2018. After obtaining verbal informed consent, members of the research team surveyed the heads of selected households with widely used and validated standardised healthcare utilisation survey questions [15, 16, 17] on demographics, socioeconomic status, and the healthcare‐seeking behaviour of household members. Healthcare‐seeking questions were asked separately about hypothetical healthcare‐seeking behaviour and actual healthcare‐seeking behaviour of individual household members and the head of the households in the event of fever <3 days, and fever ≥3 days duration. We asked the head of the household: ‘Where would household members usually seek healthcare if they had a fever of ≥3 days?’ and we collected information about the usual actual healthcare‐seeking behaviour of any individual household members experiencing a fever of ≥3 days in the past 3 months. Questionnaire choices for health seeking included YGH, other public and private hospitals, healthcare facilities in Yangon, including pharmacies, traditional healers, as well as self‐treatment, and nothing.

#### Surveillance for causes of community‐onset febrile illness

As part of the study on the aetiology of community‐onset febrile illness in Yangon, patients aged ≥12 years admitted to the Medical Observation (MO) unit of YGH during weekdays were prospectively recruited from 5 October 2015 through 4 October 2016. The methods and findings were described previously [15, 18]. Briefly, patients with an oral temperature of ≥38.0°C admitted to the MO unit were eligible for enrolment in our study. After obtaining informed consent, demographic data, including the participant's place of usual residence, were recorded. As described elsewhere [19], we collected acute serum from each participant on admission and requested all participants return 14–30 days after enrolment to provide convalescent serum. Aliquots of serum were stored at −70°C at the Department of Microbiology, University of Medicine 1, Yangon, Myanmar. Serum was shipped on dry ice to collaborating laboratories at the Mahidol Oxford Tropical Medicine Research Unit (MORU), Bangkok, Thailand.

### Laboratory methods

#### Reference serologic tests for *Orientia* and *Rickettsia*

Reference serological tests for *Orientia* and *Rickettsia* were done at MORU. Acute and convalescent samples were first screened at a dilution of 1:100 by in‐house ELISA to detect IgM and IgG antibodies against scrub typhus using Karp, Kato, Gilliam, and TA716 strains of *O. tsutsugamushi*, murine typhus using *R. typhi* Wilmington strain antigens, and against SFG using *R. honei* and *R. conorii* [12]. Whole‐cell antigens were used. A net optical density (OD) at 450 nm of ≥0.5 was used as a cut‐off for samples to proceed to indirect immunofluorescence assay (IFA) testing [12]. Samples that were positive by ELISA were then serially two‐fold titrated from 1:100 through 1:25,600 for IFA separately for *O. tsutsugamushi* using pooled Gilliam, Karp, Kato, and TA716 antigens; *R. typhi* Wilmington strain antigens; and *R. honei* and *R. conorii* antigens. The IFA was not performed on paired sera with an ELISA IgG static OD or with ELISA IgG levels above the 90th centile of all tested samples in the cohort in acute sera as these were considered likely due to antibodies present from past infection [20, 21, 22].

### Case definitions

Confirmed scrub typhus, murine typhus, and SFG were defined as a ≥4‐fold rise in IFA titre between the acute and convalescent samples [19]. Only confirmed patients who resided in the Yangon Region were included in the numerator for incidence calculations.

### Incidence calculation

**FIGURE 2:**
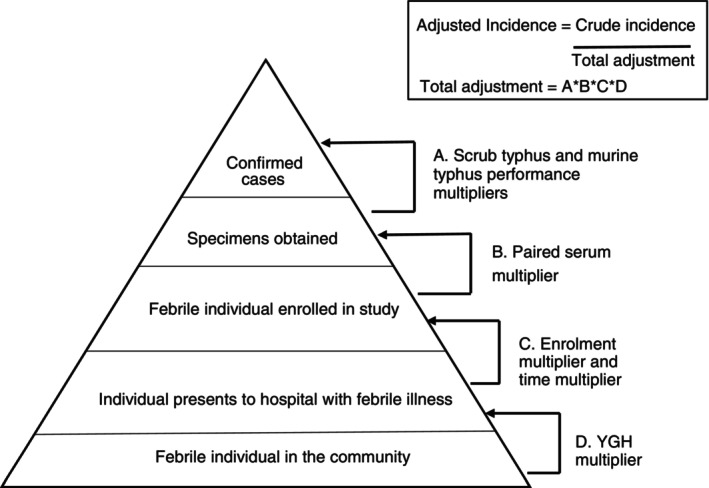
Surveillance pyramid showing multipliers used to account for incomplete case identification. Modified from Crump et al. [17] and Pisharody et al. [23]

We used multipliers from the healthcare utilisation survey and the surveillance study of causes of community‐onset febrile illness to estimate incidence. Multipliers were applied to account for febrile patients with scrub typhus, murine typhus, and SFG infections who might have been missed through the reporting process, including from healthcare provider selection, transfer from another hospital, and diagnostic test sensitivity and specificity. Multipliers were the multiplicative inverse of the relevant proportions (Figure 2). We calculated the ‘YGH multiplier’ to account for and adjust for healthcare centre choice and patients potentially missed on account of their choice of healthcare facility or options that were not monitored by our surveillance study. The ‘YGH multiplier’ was derived based on answers from heads of households to the HCUS question: ‘Where would household members usually seek healthcare if they had fever for greater than or equal to three days duration?’ We selected the first and second choice for ‘fever for greater than or equal to three days’ duration as most representative of where patients sufficiently ill to require hospital admission would seek care. We validated responses to questions about usual healthcare‐seeking questions against actual healthcare‐seeking of household members who had a fever ≥3 days in the past 3 months.

### Derivation of multipliers

The ‘time multiplier’ was calculated to account for enrolment occurring only on weekdays, five of 7 days per week. The ‘enrolment multiplier’ was derived to represent patients who were eligible but did not participate in the community‐onset febrile illness study for any reason. We calculated the referral multiplier to account for the patients transferred to YGH from other inpatient facilities, assuming that transfer may not reflect a patient's preference for a healthcare facility. The diagnostic test ‘sensitivity’ and ‘specificity’ multipliers were applied to confirmed cases to reflect the sensitivity and specificity of paired serum IFA for the diagnosis of scrub typhus, murine typhus, and SFG.

### Sensitivity analysis

Consistent with previous work [15, 16], one‐way sensitivity analysis was performed using a 95% confidence interval with the upper and lower bounds of the hospital multiplier derived from a binomial exact test for the question about healthcare‐seeking behaviour: ‘Where would household members usually seek healthcare when a household member had fever for more than or equal to 3 days?’

### Statistical analysis

Data were entered and stored in Microsoft Excel 2016 (Microsoft Corporation, Redmond, WA, USA) and analysed using STATA, version 15.1 (STATA‐Corp, College Station, TX, USA). Sensitivity analyses and incidence calculations were carried out using Microsoft Excel 2016 (Microsoft Corporation. Redmond, WA, USA). Validation of the usual healthcare seeking choice against actual health seeking behaviour was calculated by comparison of proportions using the binomial exact test in STATA, version 15.1. We also did one‐way sensitivity analysis using a 95% confidence interval with upper and lower diagnostic sensitivity multipliers.

### Research ethics

This study was approved by the Research and Ethics Review Committee of University of Medicine 1; the Department of Medical Research, Myanmar; and the University of Otago Human Ethics Committee, New Zealand.

## RESULTS

### Healthcare utilisation survey

We enrolled 336 households, including 1,598 household members. Among 336 households, 57 (16.9%) declined to participate and were replaced with another randomly selected household. Among 336 households, all had at least one member who was aged ≥20 years, 148 (44.0%) had at least one member aged from 12 through 19 years, and 144 (42.9%) had at least one member aged <12 years. Among heads of household who answered questions on usual health seeking, one (0.3%) of 336 heads of the households identified YGH as the healthcare facility that household members aged ≥12 years would seek healthcare for fever ≥3 days duration. This formed the basis of the ‘YGH multiplier’ [15].

Of 1,598 household members answering questions on actual healthcare seeking in the event of fever, 237 (14.8%) reported having fever of ≥3 days duration in the past 3 months. Of those who experienced fever during last 3 months, one (0.4%) sought care from YGH. The difference in proportion of ‘usually healthcare seeking’ against ‘actual healthcare seeking’ behaviour of the community using YGH as healthcare facility in case of fever was not statistically significant (*p* = 0.84).

### Fever surveillance study

Among 947 participants enrolled in the community onset febrile illness study, 671 (70.9%) were from Yangon Region, 170 (17.8%) were adolescents, and 777 (82.2%) were adults. Of 671 residents of the Yangon Region, 279 (41.6%) participants provided paired sera. Of those providing paired sera, 8 (2.9%) had confirmed scrub typhus, 3 (1.1%) had confirmed murine typhus, and none had confirmed spotted fever rickettsiosis.

### Multiplier derivation

During the screening for fever surveillance community onset febrile illness study, 1,045 patients were eligible for enrolment. Among 1045 eligible febrile patients, 947 (90.6%) were enrolled of which 170 (18.0%) were adolescents and 777 (82.0%) adults, resulting in an overall enrolment multiplier of 1.10. Of febrile admitted participants from Yangon Region, 22 (19.1%) of 115 adolescents and 86 (15.5%) of 556 adults have transferred from other inpatient hospitals of the region. Consequently, we calculated the referral multiplier of 0.81 and 0.85 for adolescents and adults, respectively, to adjust crude case numbers.

Based on the literature, the diagnostic sensitivity of a ≥4‐fold rise in IFA titre for murine typhus was 97.5% and specificity was 100% compared with cases confirmed by polymerase chain reaction with genome sequencing [24]. The diagnostic sensitivity of a ≥4‐fold rise in IFA titre for scrub typhus to a threshold of at least ≥1:200 was 54.0% and the specificity approaches 100% against culture‐confirmed cases [25]. We adjusted case numbers of scrub typhus by applying the paired serum IFA sensitivity and specificity multipliers of 1.85 and 1.00, respectively [25]. Murine typhus case numbers were also adjusted for paired serum IFA sensitivity and specificity by multiplying by 1.02 and 1.00, respectively [26].

Of participants residing in the Yangon Region, 279 (53.8%) of 671 provided paired sera, resulting in a ‘paired sera multiplier’ of 2.41. Among 336 households selected, one head of the household reported that they would seek healthcare at YGH in the event of fever of ≥3 days. All 336 households had at least one member aged 12 year and above whereas 148 household had at least one member who was aged 12–19 years. The resulting ‘YGH multipliers’ are shown in Table 1.

**TABLE 1:** Multipliers based on responses to relevant questions in healthcare utilisation survey, Yangon Region, Myanmar, 2018.

Age (years) of household member	Households	YGH	YGH proportion	YGH multiplier
Where household members would usually seek healthcare if they had fever of ≥3 days?				
12–19	148	1	0.68	148
≥20	336	1	0.30	336
≥12	336	1	0.30	336

Abbreviation: YGH, Yangon General Hospital.

### Incidence calculations

After applying all adjustments, the overall estimated annual incidence of scrub typhus among adolescents and adults in the Yangon Region in 2015–2016 was 211 per 100,000 persons per year, with scrub typhus incidence among adults estimated at 240 per 100,000 persons per year and incidence among adolescents was 145 per 100,000 persons per year, respectively. The overall estimated annual incidence of murine typhus among adolescents and adults in the Yangon Region in 2015–2016 was 44 cases per 100,000 persons per year, with murine typhus incidence among adults estimated at 44 per 100,000 persons per year and incidence among adolescents estimated at 40 per 100,000 persons per year. Since no SFG rickettsioses were identified, we did not estimate incidence for SFG rickettsioses. The details of incidence calculations and results are shown in Table 2.

**TABLE 2:** Estimate incidence of scrub typhus and murine typhus, Yangon Region, Myanmar 2015–2016.

	Age, years	Sex		YGH‐confirmed cases‐based paired serum	Sensitivity multiplier	Specificity multiplier	YGH multiplier	Time multiplier	Enrolment multiplier	Referral multiplier	Paired serum multiplier	Annual cases	Population	Case per 100,000 persons
		M	F		Acute and convalescent serum	Acute and convalescent serum								
Scrub typhus	≥12	4	4	8	1.85	1.00	336	1.40	1.10	0.84	2.41	15,503	7,360,703	211
	≥12–19	0	2	2	1.85	1.00	148	1.40	1.10	0.81	2.41	1,646	1,131,867	145
	≥20	4	2	6	1.85	1.00	336	1.40	1.10	0.85	2.41	11,766	4,911,502	240
Murine typhus	≥12	1	2	3	1.02	1.00	336	1.40	1.10	0.84	2.41	3,205	7,360,703	44
	≥12–19	0	1	1	1.02	1.00	148	1.40	1.10	0.81	2.41	454	1,131,867	40
	≥20	1	1	2	1.02	1.00	336	1.40	1.10	0.85	2.41	2,162	4,911,502	44

Abbreviation: YGH, Yangon General Hospital.

### Sensitivity analysis

The one‐way sensitivity analysis showed that the annual estimated incidence of scrub typhus among adolescents and adults was ranged from 39 to 7800 cases per 100,000 populations per year, and the annual estimated incidence of murine typhus ranged from 8 to 1613 cases per 100,000 populations per year (Table 3).

**TABLE 3:** Sensitivity analysis of scrub typhus and murine typhus incidence estimate, Yangon Region, 2015–1016.

	Number of cases	Multiplier (95% CI lower CI‐ upper CI)	Case per 100,000 persons
Variation in multipliers based on varying the question from the healthcare utilisation survey “Where household members would usually seek healthcare if they had fever of ≥3 days?”			
Scrub typhus	8	336 (62, 12,444)	39–7,800
Murine typhus	3	336 (62, 12,444)	8–1,613
Variations in estimation of sensitivity of IFA			
Confirmed cases of scrub typhus	8	1.85 (1.56, 2.32)	178–264
Confirmed cases of murine typhus	3	1.02 (1.01, 1.09)	43–47

Abbreviation: IFA, indirect immunofluorescent antibody test.

## DISCUSSION

We found that the estimates of annual incidence of scrub typhus among adolescents and adults were high and similar to adolescent and adult typhoid incidence estimates for the Yangon Region [15], whereas murine typhus incidence was lower. To our knowledge, ours are the first estimates of the incidence of scrub typhus and murine typhus in the Yangon Region, Myanmar. No confirmed SFG rickettsioses were identified in our study.

Since our estimates are of illnesses sufficiently severe to require hospitalisation, we are likely missing disease in patients seeking care in outpatient settings or remaining at home. Alternatively, fever of 3 days or more duration may be a poor surrogate for illness sufficiently severe to seek healthcare at a hospital, potentially contributing to an overestimation of disease incidence. Since few participants in the Yangon Region use YGH as the first or second source of care for prolonged fever, the uncertainty around our incidence estimates was wide. Our incidence estimates were considerably higher than those published from the Republic of Korea or Thailand. However, those studies did not attempt to adjust for health seeking and other factors, so may have underestimated incidence [27, 28].

In Laos and Thailand, scrub typhus and murine typhus account for up to 28% of febrile admissions [8, 29, 30]. Furthermore, scrub typhus epidemiology is heterogeneous at the sub‐national level, with marked seasonality and differences between rural and urban settings. In Thailand, the highest incidence of scrub typhus was observed in the north and accounted for approximately half of all scrub typhus in Thailand despite being home to just 18.6% of the national population [27]. Similarly, seroprevalence studies in Laos suggest that scrub typhus is more prevalent in rural settings than in urban areas, whereas murine typhus is more common in urban settings [31]. A separate analysis of risk factors for scrub typhus in the present Myanmar study showed that living rurally and being an agricultural worker were associated with increased odds of scrub typhus [19]. Given sub‐national variation in rickettsial disease epidemiology [27], we suggest that further research elsewhere in Myanmar would be useful to inform management, prevention, and control. While we found no confirmed SFG rickettsioses in the Yangon Region, others reported that SFG antibodies were present among 3% of participants from seven distinct locations in Myanmar [12].

Murine typhus can be acquired at home through contact with rats that act as reservoirs with transmission via associated flea vectors [32]. The risk for murine typhus increases with rodent abundance [32]. In a survey of slaughterhouse workers from the Yangon Region, 61.2% reported seeing rodents in their homes [33]. A range of rodent species may serve as hosts for *R. typhi*, acting as vertebrate amplifying hosts, and *O. tsutsugamushi*, acting as dead‐end hosts [32, 34]. A separate analysis of risk factors for murine typhus exposure in the present Myanmar study showed that living rurally was associated with reduced odds of murine typhus exposure [19].

The combination of sentinel hospital surveillance with healthcare utilisation studies has been used successfully in resource‐limited settings to estimate the incidence of a range of acute infectious illnesses [16, 17]. In Myanmar, such hybrid surveillance approaches have been used to assess the incidence of enteric fever [15]. While our findings represent our best effort to estimate scrub typhus, murine typhus, and SFG rickettsioses incidence data for the Yangon Region, we acknowledge that there are several limitations. Because resource constraints and logistical challenges prevented active surveillance of the entire population, we chose to estimate incidence using the multiplier or hybrid surveillance approach [17, 35]. Although this method of incidence estimation is widely accepted, our estimates are based on a limited number of illnesses, and so some of the variation could be due to random error. Furthermore, the multiplier method is predicated on several assumptions: that the same proportion of cases occurred among those who did not have paired sera collected compared to those who had paired sera available for testing; that eligible but non‐enrolled patients had the same prevalence of acute scrub typhus and acute murine typhus as those who were enrolled; those presenting to surveillance sites were representative of the respondents enrolled in the healthcare utilisation survey; that survey questions accurately capture healthcare seeking practices in the catchment; and that usual healthcare seeking practices represent actual healthcare seeking practices. Due to the lack of a sentinel surveillance study for community‐onset febrile illness at paediatric healthcare facilities, we were unable to estimate incidence in that key age group. Since there is presently no means to reliably interpret single acute sample serology for rickettsial diseases, we could not accurately diagnose these infections in participants without paired sera [26, 36]. Unfortunately, few households reported using YGH as their first or second choice for prolonged febrile illness, resulting in very wide uncertainty associated with our incidence estimates.

## CONCLUSION

We provide the first estimates of scrub typhus and murine typhus incidence in Myanmar, demonstrating high incidence of scrub typhus and medium incidence of murine typhus among adolescents and adults in the Yangon Region. Further research is needed to describe other components of disease burden in Myanmar, including illness duration, complications, and death. Research in other geographic areas of Myanmar would help to understand variation in the distribution of disease nationwide. Involving younger age groups would improve understanding of variation in incidence by age. Our study was done in parallel with research to identify risk factors for infection that should be used to inform prevention and control of scrub typhus and murine typhus in Myanmar [19]. The relatively high incidence estimates suggest that these diseases warrant policy attention in Myanmar for vector‐borne disease control.

## CONFLICT OF INTEREST STATEMENT

The authors declare no conflicts of interest.
